# Ferromagnetism in exfoliated tungsten disulfide nanosheets

**DOI:** 10.1186/1556-276X-8-430

**Published:** 2013-10-17

**Authors:** Xingze Mao, Yan Xu, Qixin Xue, Weixiao Wang, Daqiang Gao

**Affiliations:** 1 Key Laboratory for Magnetism and Magnetic Materials of MOE, Lanzhou University, Lanzhou 730000, People’s Republic of China; 2 Physics Department, Xinxiang University, Xinxiang 453003, People’s Republic China

**Keywords:** WS2, ferromagnetism, nanosheet

## Abstract

Two-dimensional-layered transition metal dichalcogenides nanosheets have attracted tremendous attention for their promising applications in spintronics because the atomic-thick nanosheets can not only enhance the intrinsic properties of their bulk counterparts, but also give birth to new promising properties. In this paper, ultrathin tungsten disulfide (WS_2_) nanosheets were gotten by liquid exfoliation route from its bulk form using dimethylformamide (DMF). Compared to the antiferromagnetism bulk WS_2_, ultrathin WS_2_ nanosheets show intrinsic room-temperature ferromagnetism (FM) with the maximized saturation magnetization of 0.004 emu/g at 10 K, where the appearance of FM in the nanosheets is partly due to the presence of zigzag edges in the magnetic ground state at the grain boundaries.

## Background

Together with the rapidly increasing research interests on graphene and their devices in the last few years, inorganic-layered structure materials, such as tungsten disulfide (WS_2_) and MoS_2_ also attracted extensive attention because of their unique physics properties [1-5]. Similar to graphite, such layered structure materials crystallize in a van der Waals-layered structure where each layer consists of a slab of S-X-S (*X* = W, Mo) sandwich. MoS_2_ monolayers have been isolated via mechanical exfoliation, wet chemical approaches, physical vapor deposition, and sulfurization of molybdenum films [6-9]. At the same time, their electronic, optical, and magnetic properties including carrier mobilities of approximately 200 cm^2^V^−1^s^−1^, photoluminescence, and weak room temperature ferromagnetism have been proposed [1-5,10,11]. So far, MoS_2_ has been explored in diverse fields and integrated in transistors and sensors, and used as a solid-state lubricant and catalyst for hydrodesulfurization, hydrogen evolution, and so on [6-9,12,13].

Recently, mechanically exfoliated, atomically thin sheets of WS_2_ were also shown to exhibit high in-plane carrier mobility and electrostatic modulation of conductance similar to MoS_2_[14,15]. Differential reflectance and photoluminescence spectra of mechanically exfoliated sheets of synthetic 2H-WS_2_ with thicknesses ranging between 1 and 5 layers were also reported, where the excitonic absorption and emission bands were found as gradually blue shifted with decreasing number of layers due to geometrical confinement of excitons [16]. Gutiérrez et al*.* described the direct synthesis of WS_2_ monolayers via sulfurization of ultrathin WO_3_ films with triangular morphologies and strong room-temperature photoluminescence [17], which could be used in applications including the fabrication of flexible/transparent/low-energy optoelectronic devices.

Even though the electrical, mechanical, and optical properties of WS_2_ have been studied both theoretically and experimentally, recent studies on the magnetic response of WS_2_ are limited. Murugan et al*.* revealed by first-principles calculations that stoichiometric Mo_*n*_S_2*n*_ (*n* = 1, 2, 5, and 6) and W_6_S_12_ clusters as well as several of the nonstoichiometric clusters are magnetic, where the magnetic moments arise due to the partially filled *d* states [18]. Besides, calculation results indicate that adsorption of nonmetal elements on the surface of WS_2_ nanosheets can induce a local magnetic moment [19]. In an experimental study, Matte et al*.* fabricated WS_2_ nanosheets by hydrothermal method and revealed their ferromagnetism, which was considered to be related to the edges and defects [20].

Developed liquid exfoliation process is considered to be an effective pathway to prepare the ultrathin two-dimensional nanosheets of intrinsically layered structural materials with high quality [21]. In this paper, the ultrathin WS_2_ nanosheets were gotten by exfoliating bulk WS_2_ in *N*,*N*-dimethylformamide (DMF, 100 mL) solution as in our previous report [22], and we studied the magnetic properties of WS_2_ nanosheets experimentally from 300 K down to 10 K. Results indicate that the fabricated WS_2_ nanosheets show clear room-temperature ferromagnetism which possibly originates from the existence of zigzag edges or defects with associated magnetism at grain boundaries.

## Methods

WS_2_ nanosheets were prepared through exfoliating of bulk WS_2_. In a typical synthesis progress, 0.5 g of WS_2_ powders was sonicated in N, N-Dimethylformamide (DMF, 100 mL) to disperse the powder. After precipitation, the black dispersion was centrifuged at 2000 rpm for about 20 minutes to remove the residual large-size WS_2_ powders. Then, the remainder solution was centrifuged at 10000 rpm for 1 h to obtain the black products. To remove the excess surfactant, the samples were repeatedly washed with ethanol and centrifuged. Finally, the samples were dried at 60°C in vacuum condition.

## Results and discussion

**Figure 1:**
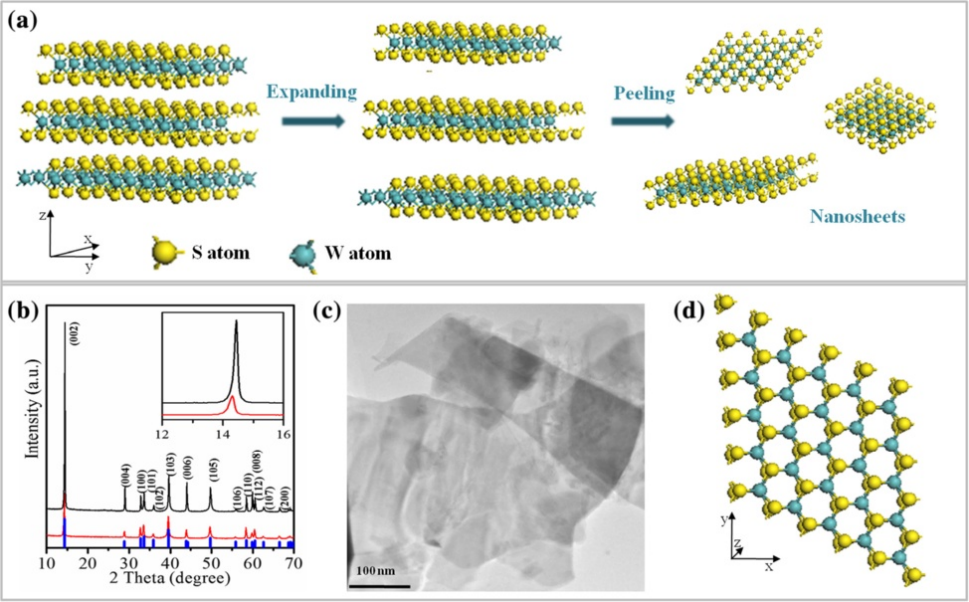
**Schematic illustration of liquid exfoliation process, XRD results, TEM, and theoretically perfect crystal structure of WS**_**2**_**. (a)** Schematic illustration of liquid exfoliation process from bulk WS_2_ to ultrathin nanosheets. **(b)** XRD results for pristine WS_2_ bulk (black line) and the exfoliated nanosheets (red line), the blue line is the standard WS_2_ diffraction peaks got from JCPDS card no. 85-1068. **(c)** TEM image of the exfoliated WS_2_ nanosheets. **(d)** A theoretically perfect crystal structure of the single-layered WS_2_.

Figure 1a shows the schematic illustration of liquid exfoliation process from bulk WS_2_ to ultrathin nanosheets. When ultrasonication was carried out in the DMF solution, the WS_2_ bulk materials swelled with the insertion of DMF molecules into the layers, which can then be easily exfoliated into the nearly transparent ultrathin nanosheets. In the absence of any high-temperature treatment or oxidation process, the exfoliated nanosheets will retain the same crystal structure of the bulk materials. Typical X-ray diffraction (XRD, X' Pert PRO Philips with Cu Kα radiation; Philips, Anting, Shanghai, China) patterns of the WS_2_ bulk and nanosheets are reported in Figure 1b. During the XRD test, the exfoliated WS_2_ nanosheets were collected together onto the glass substrate. That is to say, the XRD result can be gotten just as the other powder sample in our case. It can be seen that all the diffractions for the exfoliated nanosheets are corresponding to the hexagonal phase of WS_2_ (JCPDS card no. 85-1068) and as comparable to the bulk form. The dominated (002) diffraction peak indicates the growth of WS_2_ along the *c*-axis direction. Results indicate that the (002) peak for the exfoliation nanosheets decreased in intensities when compared to the pristine WS_2_, and the notable shift to the low intensity was also observed, which may be caused by the decrease of structural crystalline and the increasing of the disorder or defect density [23]. Figure 1c shows the transmission electron microscopy (TEM, TecnaiTM G2 F30, FEI, Hillsboro, OR, USA) image of the exfoliated product, from which one can see that the free-standing nanosheets were inhomogenous with different sizes and morphologies.

**Figure 2:**
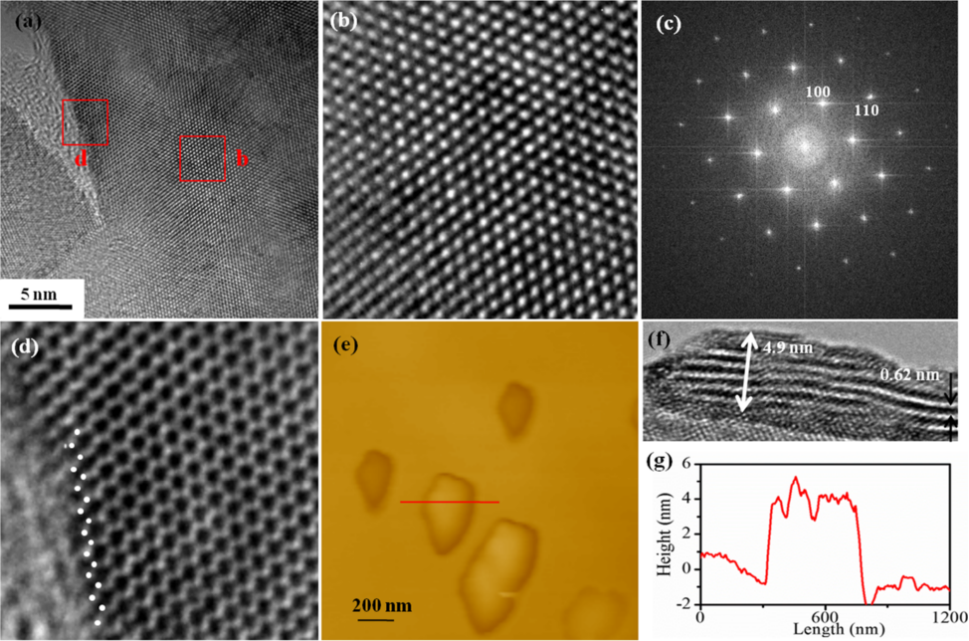
**Different types of imaging showing different characteristics of formed WS**_**2 **_**nanosheets and FFT analysis. (a)** TEM image of the WS_2_ nanosheets. **(b, d)** High-resolution TEM images for the selected regions are shown. **(c)** Two-dimensional FFT analysis for the WS_2_ nanosheets. **(e)** Tapping-mode AFM image of the WS_2_ nanosheets and **(g)** the corresponding thickness distribution. **(f)** High-resolution TEM image of the curled edge for the nanosheets.

High-resolution TEM (HRTEM) image and the two-dimensional fast Fourier transform (FFT) analysis (Figure 2b,c) reveal the hexagonal lattice structure with the lattice spacing of 0.27 and 0.16 nm assigned to the (100) and (110) planes [17]. Further high-resolution TEM results for the selected regions for the inner and the edges of one nanosheet are shown in Figure 2b,d, respectively. Results indicate that the inner part of the nanosheets has a well-crystallographic structure without existence of defects. On the contrary, a clear disorder is observed at the edges; the result reveals a hexagonal arrangement of atoms with zigzag edges. The size distribution of as-prepared WS_2_ nanosheets was evaluated from the tapping-mode atomic force microscopy (AFM Dimension 3100 with Nanoscope IIIa controller, Veeco, CA, USA). As can be seen from Figure 2e, the diameter of the nanosheets ranges from 200 to 500 nm, in accordance with the TEM observation. As also shown in Figure 2e, the randomly measured thicknesses for the nanosheets are ranging from 1.2 to 4.8 nm, where the maximum height profile of 4.9 nm is shown in Figure 2g. Considering that the *c* parameter of WS_2_ is 0.62 Å, the thickness of 1.8 to 4.9 nm denoted that the nanosheets comprised 2 ~ 8 single layers of WS_2_. Accidentally, some WS_2_ nanosheets have curled edges, rendering it possible to evaluate a sheet thickness during high-resolution TEM. One can see from Figure 2f that the nanosheet with 3 ~ 8 layers thick shows the presence of a high density of edges. Besides, the clear bend can be observed, which may arise from defects at the edges.

**Figure 3:**
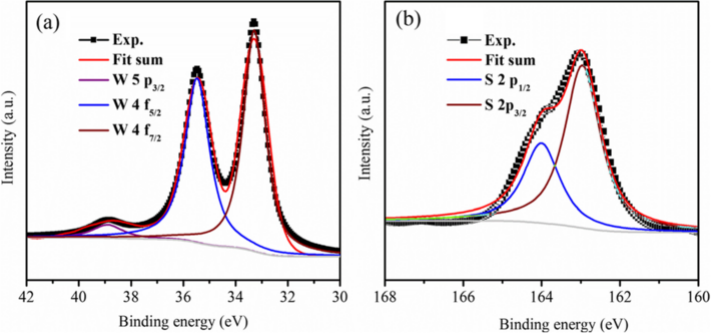
**High-resolution XPS scan of (a) W 5p and W 4f**, **(b) S 2p for WS**_**2 **_**nanosheets.**

The bonding characteristics and the composition of the WS_2_ nanosheets were captured by X-ray photoelectron spectroscopy (XPS, VG ESCALAB 210; Thermo Fisher Scientific, Hudson, NH, USA), where the standard C 1*s* peak was used as a reference for correcting the shifts. Results indicate that there only W, S, and C elements are detected in the XPS survey. The peaks shown in Figure 3b, corresponding to the S 2*p*_1/2_ and S 2*p*_3/2_ orbital of divalent sulfide ions, are observed at 163.3 and 162.1 eV. Besides, the W peaks shown in Figure 3a located at 38.9, 35.5, and 33.3 eV are corresponding to W 5*p*_3/2_, W 4*f*_5/2_, and W 4*f*_7/2_, respectively. The energy positions of these peaks indicate a W valence of +4, which is in accordance with the previous reports, indicating the formation of pure WS_2_ phase [24].

**Figure 4:**
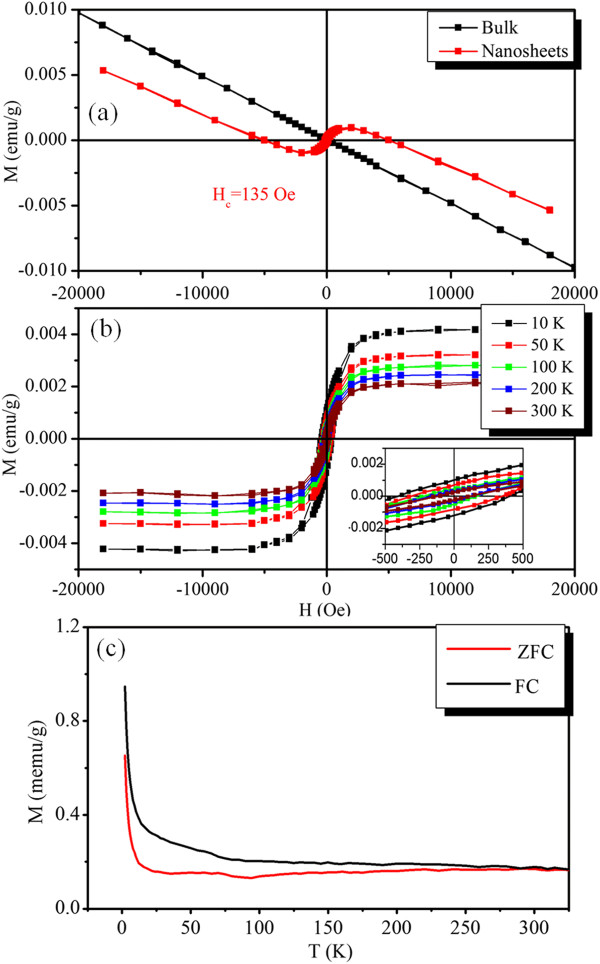
**M-H curves for pristine WS**_**2 **_**bulk and nanosheets and FC and ZFC curves for WS**_**2 **_**nanosheets. (a)** The M**-**H curves for the pristine WS_2_ bulk and the WS_2_ nanosheets.** (b)** M-H curves for the WS_2_ nanosheets measured at different temperatures, where the diamagnetic signal has been deduced. **(c)** The FC and ZFC curves for the WS_2_ nanosheets.

Single crystals of the bulk WS_2_ are expected to be diamagnetic just like any other semiconductors, which is confirmed by the measured magnetization versus magnetic field (*M*-*H*) curve shown in Figure 4a using the Quantum Design MPMS magnetometer (Quantum Design, Inc, San Diego, CA, USA) based on superconducting quantum interference device (SQUID). However, for the WS_2_ nanosheets, even though the magnetic response is dominated by the diamagnetism, it is found that the diamagnetic background is superimposed onto the ferromagnetic loop, implying that the total magnetic susceptibility comprises both diamagnetic and ferromagnetic parts (shown in Figure 4a). After subtracting out the diamagnetic part, the ferromagnetic response at different temperatures has been plotted in Figure 4b. The clear S-shaped saturated open curves at all the measured temperatures with the saturation magnetization (*M*_s_) of 0.002 emu/g at room temperature are observed, revealing the room-temperature ferromagnetism (FM) nature of the WS_2_ nanosheets. In addition, one can observe that the *M*_s_ and the coercivity (*H*_c_) decrease as the temperature increases from 10 to 330 K, revealing a typical signature of nominal FM-like material. The temperature-dependent magnetization measurements for WS_2_ nanosheets recorded at 100 Oe are shown in Figure 4c. The first measurement was taken after zero-field cooling (ZFC) to the lowest possible temperature (2 K), and in the second run the measurements were taken under field-cooled (FC) conditions. When cooling down from 330 K, both the ZFC and FC data follow similar trend, that is, slow increase of susceptibility until 40 K followed by a sharp rise. Note that the two curves are separated in the whole measured temperature ranges, revealing that the Curie temperature of the sample is expected to exceed 330 K.

Recently, similar ferromagnetic nature was also observed in other layered materials, like graphene, graphene nanoribbons, and MoS_2_. Matte et al*.* and Enoki et al*.* proposed that edge states as well as adsorbed species affect the magnetic properties of graphene [25,26]. Zhang et al*.* prepared MoS_2_ samples with high density of prismatic edges and showed them to be ferromagnetic at room temperature, where the magnetism arising from nonstoichiometry of the unsaturated Mo and S atoms at the edge [27]. Our previous results indicate that the saturation magnetizations of the exfoliated MoS_2_ nanosheets increase as the lateral size decreases, revealing the edge-related ferromagnetism [22]. Density functional calculations on inorganic analog of graphite MoS_2_ reveal that edge states are magnetic and it appears that magnetism originates at the sulfur-terminated edges due to the splitting of metallic edge states at the Fermi level [28]. Besides, calculation results indicate that only MoS_2_-triple vacancy created in a single-layer MoS_2_ can give rise to a net magnetic moment [29]. Shidpour et al*.* indicated that a vacancy on the S-edge with 50% coverage intensifies the magnetization of the edge of the MoS_2_ nanoribbon, but such a vacancy on S-edge with 100% coverage causes this magnetic property to disappear [30]. Furthermore, MoS_2_ and WS_2_ clusters (Mo_6_S_12_ and W_6_S_12_) were shown to be magnetic, where the magnetism arising from the unsaturated central metal atom is due to partially filled *d* orbitals [18]. In our case, the WS_2_ nanosheets with 2 ~ 8 layers thick and the presence of the high density of edges can be seen from the images in Figure 2f. The bends in the layers may arise from the defects. Besides, the high-resolution TEM image of the nanosheets shown in Figure 2d reveals a hexagonal arrangement of atoms with zigzag edges. Such defective centers and edges would be associated with the W atoms, which are undercoordinated, resulting in partially filled *d* orbitals. A high concentration of such edges and defects in our samples could be one of the possible reasons for the observation of ferromagnetism.

## Conclusions

In summary, even though the observed ferromagnetism in WS_2_ is in the bulk limit, results indicate that the ferromagnetism for exfoliated WS_2_ nanosheets persists from 10 K to room temperature. We attribute the existence of ferromagnetism partly to the zigzag edges and the defects in our samples. This unusual room-temperature ferromagnetism, which is an intrinsic feature similar to that observed in carbon-based materials, may open perspectives for spintronic devices in the future.

## Competing interests

The authors declare that they have no competing interests.

## Authors’ contributions

DG participated in all of the measurements and data analysis, and drafted the manuscript. YX conceived and designed the manuscript. XM and QX prepared all the samples, carried out the XPS measurements and data analysis. WW participated in the SQUID measurements. All authors have been involved in revising the manuscript and read and approved the final manuscript.
